# Long-term effectiveness of thymectomy in late-onset myasthenia gravis

**DOI:** 10.1007/s00415-025-13424-2

**Published:** 2025-10-21

**Authors:** Ester Latini, Melania Guida, Carmelina Cristina Zirafa, Stefania Rizzo, Alba Cepele, Gaetano Romano, Vittorio Aprile, Marco Lucchi, Gabriele Siciliano, Roberta Ricciardi, Michelangelo Maestri Tassoni

**Affiliations:** 1 Present Address: Department of Experimental and Clinical Medicine, Division of Geriatric and Intensive Care Medicine, University of Florence, Viale Brambilla, 3, Firenze, Italy; 2 Department of Clinical and Experimental Medicine, Neurology Unit, University of Pisa, Via Roma 55, Pisa, Italy; 3 Minimally Invasive and Robotic Thoracic Surgery-Surgical, Medical, Molecular and Critical Care Pathology Department, University Hospital of Pisa, Pisa, Italy; 4 Neurology Unit, ASL Salerno, Agropoli, Italy; 5 Division of Thoracic Surgery, Department of Surgical, Medical and Molecular Pathology and Critical Area, University of Pisa, Pisa, Italy

**Keywords:** Late onset myasthenia gravis, Thymectomy, Myasthenia gravis, Anti-AChR Ab, Generalized myasthenia gravis, Thymic hyperplasia, Thymic atrophy, Very late onset myasthenia gravis

## Abstract

**Background:**

Thymectomy is a well-established treatment in anti-AChR generalized myasthenia gravis (gMG) patients aged 18–50 years. However, the MGTX trial failed to prove an additional benefit of thymectomy in late-onset MG (LOMG) patients and studies conducted so far have shown controversial results. The primary aim of this study was to assess the safety and effectiveness of thymectomy in LOMG patients compared to medical therapy alone.

**Methods:**

This was an observational retrospective case–control study of LOMG patients followed at the MG Clinic of Pisa University Hospital from 1996 to 2024. Inclusion criteria were: diagnosis of non-thymomatous gMG with anti-AChR antibodies; age at onset ≥ 50 years, and minimum follow-up of 12 months. The cumulative incidence of disease remission between the thymectomy and the conservative group was compared with Kaplan–Meier analysis with log-rank test and the Cox regression model was used to estimate the effect of thymectomy on achieving remission after adjustment for confounding variables.

**Results:**

Among our population of 127 LOMG patients, 87 patients underwent thymectomy, while 40 patients received medical treatment only. When evaluating neurological outcomes at the last follow-up, the thymectomy group had a 3.25-fold (HR = 3.25, 95% CI 1.31–8.1) increased probability of achieving disease remission than the conservative group, after adjustment for confounding variables.

**Conclusion:**

Our findings suggest that thymectomy may be a feasible and potentially beneficial therapeutic option in this MG subgroup, possibly increasing the likelihood of disease remission without ongoing immunosuppressive therapy.

**Supplementary Information:**

The online version contains supplementary material available at 10.1007/s00415-025-13424-2.

## Introduction

Myasthenia gravis (MG) comprises different subtypes of disease, each with specific clinical–immunological characteristics and distinct therapeutic needs. The distinction into subgroups is based on antibody pattern, thymic pathology, and clinical features. Within the subtypes associated to anti-acetylcholine receptor antibodies (anti-AChR Ab) without thymoma, age at onset allows for the differentiation between early-onset MG (EOMG) and late-onset (LOMG) MG [1]. LOMG is defined as MG that begins after the age of 50 years old [1, 2] and it has become so far the largest MG subgroup [3–6] The evolving epidemiology of MG, with an increasing incidence of MG in adults over 50 years of age, raises the question of whether a different therapeutic approach is necessary and further evidence is required to address the therapeutic unmet needs of this population.

When treating non-thymomatous anti-AChR Ab positive MG, thymectomy should always be considered and it is recommended [7, 8] given the role thymic abnormalities play, particularly thymic hyperplasia, in the pathogenesis of this subtype [9]. However, the therapeutic value of thymectomy in LOMG has not yet been established.

The MGTX trial allowed patients to participate up to the age of 65 years, but it failed to demonstrate a beneficial effect of thymectomy in LOMG compared to medical therapy alone because the small number of LOMG patients included [10, 11]. Since then, only three retrospective studies have directly compared surgical treatment to medical therapy alone in LOMG yielding conflicting results [12–14]. Even the most recent meta-analyses on the effectiveness of thymectomy in LOMG patients [15, 16] reported inconclusive findings, partly because of the heterogeneity of study cohorts and assessment methods.

As a result, international guidelines generally recommend thymectomy in non-thymomatous anti-AChRAb -positive gMG patients aged 18–50 years [8, 17–19]. Only the recent German and European Nordic countries guidelines on MG have raised the upper age limit for thymectomy to 65 years [20, 21].

Given these contradictory findings, further investigation is needed to provide clearer indications for surgery and develop a personalized strategy based on each patient’s benefit–risk profile.

The primary aim of the present study was to assess the efficacy and safety of thymectomy in patients with generalized LOMG. The neurological outcomes of the study population were compared with those of LOMG patients treated with medical therapy alone and with those of a group of EOMG patients who underwent thymectomy. Finally, among the collected data, we sought to identify demographic, clinical, and histological factors that could predict the outcome of LOMG patients.

## Methods

### Study design

This is a single-center observational case–control retrospective study. Ethical approval was granted by Local Ethical Committee CEAVNO (ID 19211) and the study was conducted according to the Declaration of Helsinki and its amendments.

### Inclusion and exclusion criteria

We retrospectively analyzed the clinical records of a cohort selected from the population of non-thymomatous LOMG patients followed at the MG Clinic of Azienda Ospedaliero-Universitaria Pisana from 1996 to 2024.

The cohort was selected according to the following inclusion criteria: (1) diagnosis of MG based on typical clinical manifestation and/or electrophysiological testing (single-fiber electromyography or repetitive nerve stimulation) demonstrating a postsynaptic neuromuscular junction disorder [18]; (2) positive anti-AChR antibodies; (3) age at disease onset greater than 50 years; (4) exclusion of a malignant thymic involvement by chest imaging and/or histological examination after surgery (5) a minimum follow-up period after thymectomy or baseline (T0) of 12 months. Patients with anti-MuSK, anti-LRP4 antibodies, or triple seronegative MG were excluded. Patients whose symptoms did not generalize within three years from onset were also excluded.

Among the LOMG cohort, patients who underwent thymectomy were designated as the “thymectomy group” and those who received medical treatment only were designated as “the conservative group”. Patients with an age at onset > 65 years were classified as having very-late-onset MG (VLOMG) according to latest evidence [5, 22]. We also identified a control group of 87 adult patients with generalized anti-AChR-Ab-positive EOMG who underwent thymectomy between 2012 and 2022, to compare their neurological outcomes with those of the LOMG thymectomy group.

For each of the 127 LOMG patients identified, we reported demographics and clinical features at baseline (T0), including sex, age at onset, symptoms at onset and comorbidities. For those who underwent thymectomy, we also reported age at surgery, timing of surgery from disease onset in months, surgical approach [sternotomy or minimally invasive robotic approach], post-operative complications, and histopathological results. Preoperatively, radiological evaluation of the mediastinum was performed in all patients using chest CT scans, and findings were reviewed through multidisciplinary board discussions. To avoid the time bias known as guarantee-time bias or immortal time bias [23], the baseline evaluation (T0) for LOMG patients in the conservative group was set at 29 ± 11 months (mean ± SD) from disease onset, corresponding to mean timing of thymectomy since disease onset in the thymectomy group.

The severity of disease at onset was classified according to Myasthenia Gravis Foundation of America (MGFA) classification [24].

At each visit, neurological evaluation was conducted using the Myasthenia Gravis Activities of Daily Living (MG-ADL) and the Myasthenia Gravis Composite scale (MGC), incorporating both patient-reported outcomes measures (PROMs) and clinician-reported ones (CROMs), respectively [25–27].

At each evaluation, we also recorded ongoing MG treatments, including the daily dosage of prednisone, immunosuppressive medications (such as Azathioprine, Mycophenolate Mofetil and Cyclosporine), and the need for rescue therapy (IVIg/PLEX) for MG relapses.

The neurological outcome at the last available follow-up was assessed using the Myasthenia Gravis Foundation of America Post-Intervention Status (MGFA-PIS) [24].

The prognosis was considered favorable if the MGFA-PIS indicated complete stable remission (CSR) or pharmacologic remission (PR). CSR was defined as the absence of any symptoms or signs of MG for at least one year without any treatment for MG. PR was defined similarly as no symptoms or signs of MG for at least one year, but with ongoing therapy for MG other than cholinesterase inhibitors. For each patient who achieved CSR or PR, the time to disease remission, expressed in months from T0, was also recorded. In addition to disease remission (CSR and PR), achieving minimal manifestation status (especially MM-1 state) was regarded as a positive outcome. We also assessed disease severity at the last follow-up using the MGFA classification, in accordance with a recent consensus statement that allows a score of zero to be assigned to asymptomatic patients [28].

Surgical approach selection was at the discretion of the operating surgeon; nonetheless, the minimally invasive technique has emerged as the most common approach since 2015.

### Statistical analysis

Continuous data were presented as mean (standard deviation) or median (interquartile range) depending on whether the data showed normal distribution, and the datasets were analyzed using student's *t* test, one-way ANOVA or Mann–Whitney/Kruskal–Wallis where appropriate. The categorical data were presented as numbers or proportions (%) and comparisons were assessed by either chi-Square test or Fisher's exact test.

Kaplan–Meier analysis with log-rank test was performed to compare the cumulative incidence curve of PR, CSR, and disease remission (CSR + PR) between the patients who underwent thymectomy and those who received only medical treatment.

The Cox proportional hazards regression model was used to estimate the effect of thymectomy on achieving disease remission (CSR and PR) after adjustment for age, sex, disease severity, and immunosuppressive therapy.

Logistic regression was performed to evaluate and identify predictors of disease remission in the entire population adjusting for age at onset, sex, disease severity at onset, and at baseline. *P* values were considered significant when < 0.05. All statistical analyses were performed with IBM SPSS Statistics (version 29.0).

## Results

We identified a total of 127 LOMG patients: 87 patients underwent thymectomy (thymectomy group), while 40 patients received only medical treatment (conservative group). Median age at onset was 59 (11) years [median (IQR)], and 32 patients were female (25.2%), with a female-to-male ratio of 1:3. The clinical characteristics of the two groups at baseline are displayed and compared in Table 1.

**Table 1:** Comparison of baseline characteristics of thymectomy and conservative LOMG cohorts

	Thymectomy group *n *= 87	Conservative group *n* = 40	*p* value*
Sex n (%)			
Female	22 (25.3%)	10 (25%)	ns
Male	65 (74.7%)	30 (75%)	
Age at onset median (IQR)	56.5 (10) years	59.5 (10) years	**0.009**
MGFA classification at onset n (%)			
1	9 (10.3%)	0	**0.035**
2A	13 (14.9%)	4 (10%)	ns
2B	31 (35.6%)	16 (40%)	ns
3A	2 (2.3%)	1 (2.5%)	ns
3B	24 (27.6%)	10 (25%)	ns
4B	7 (8%)	7 (17.5%)	ns
5	1 (1.1%)	2 (5%)	ns
MG treatment at baseline n (%)			
Prednisone	83 (95.4%)	37 (92.5%)	ns
Immunosuppressants	11 (12.6%)	13 (32.5%)	**0.008**
Dose of prednisone at baseline median (IQR)	25 (24.06) mg/die	17.5 (18.75) mg/die	**< 0.001**
IVIG/PLEX at baseline n (%)	21 (24.1%)	13 (32.5%)	ns
MG ADL at baseline median (IQR)	5 (3)	4 (4)	ns
MGC score at baseline median (IQR)	7 (5)	6 (6)	ns
Comorbidities n (%)			
DM	19 (21.8%)	13 (32.5%)	ns
CAD	5 (5.7%)	6 (15%)	ns
Cerebrovascular disease	6 (6.9%)	4 (12.5%)	ns
AF	3 (3.4%)	2 (5%)	ns
Hypertension	42 (48.3%)	27 (67.5%)	0.055
COPD	5 (5.7%)	2 (5%)	ns
Osteoporosis	18 (20.7%)	6 (15%)	ns
Extra-thymic malignancy	10 (11.5%)	9 (22.5%)	ns
Autoimmune disease	16 (18.3%)	19 (47.5%)	**< 0.001**

Data are shown as mean ± SD or median (IQR) depending on whether the data showed normal distribution

*LOMG* Late-onset myasthenia gravis, *MGFA* Myasthenia Gravis Foundation of America, *MG ADL* Myasthenia gravis activities of daily living, *MGC score* Myasthenia gravis composite score, *IVIg* Intravenous immunoglobulin, *PLEX* Plasma exchange, *SD* Standard deviation, *IQR* Interquartile range, *DM* Diabetes mellitus, *CAD* Coronary artery disease, *AF* Atrial fibrillation, *COPD* Chronic obstructive pulmonary disease

^*^Chi-square test for categorical variables, Mann–Whitney test for continuous variables

Age at onset was younger in the thymectomy group (*p* = 0.009) and the thymectomy group had a higher proportion of pure ocular manifestations at onset (MGFA class I; *p* = 0.035). Furthermore, the two groups differed at baseline in terms of MG medications. Immunosuppressants were used more frequently in the conservative group (*p* = 0.008), while the median dosage of steroid therapy was higher in the thymectomy group (*p* =  < 0.001).

No differences were instead observed in terms of sex, disease severity, as assessed by MG-ADL and MGC scales at baseline, or comorbidities, such as hypertension, diabetes mellitus, coronary artery disease, cerebrovascular disease, and extra-thymic malignancy. Notably, only autoimmune diseases had a different distribution in the two groups, with a higher rate in the conservative group (*p* = 0.003).

The most common medical conditions in the entire cohort were hypertension (*n* = 69), autoimmune diseases (*n* = 35), diabetes mellitus (*n* = 32), osteoporosis (*n* = 24), and extra-thymic malignancies (*n* = 19). Cardiovascular burden was higher in the conservative group although this difference did not reach statistical significance. Thirty-five autoimmune diseases were associated with MG, with Hashimoto’s thyroiditis being the most common (*n* = 26), followed by psoriatic arthritis (*n* = 4) and autoimmune atrophic gastritis (*n* = 2). As above-mentioned, the distribution of autoimmune diseases between the conservative and thymectomy groups was different; however, this did not influence the use of immunosuppressants, which were primarily prescribed for MG control in both groups.

Histological findings and clinical features related to surgery of the 87 patients in the thymectomy group are presented in Table 2. Most patients underwent thymectomy within 22 (12–75) months [median (range)] from disease onset and at a median age of 59 (51–79) years [median (range)]. The procedure was safe in most cases with few post-operative complications: one myasthenic crisis successfully treated with IVIg and no requirement for ventilatory support, as well as other complications primarily related to the surgical procedure, such as subcutaneous emphysema and pleural effusion.

**Table 2:** Surgical features of the thymectomy group (n = 87)

Age at surgery median (range)	60 (51–79) years
Timing of thymectomy from onset median (range)	21.5 (3–156) months
Surgical approach n (%)	
Robotic surgery	64 (73.6%)
Sternotomy	23 (26.4%)
Postoperative complications n (%)^§^	6 (6.8%)
Histology n (%)	
Thymic hyperplasia	31 (35.6%)
Thymic atrophy/involution	45 (51.7%)
Thymic cyst	7 (8%)
Normal residual thymic tissue	3 (3.4%)
Cavernous hemangioma	1 (1.1%)

^**§**^ Postoperative complications recorded were as follows: myasthenic crisis (1), paroxysmal atrial fibrillation (1), post-operative subcutaneous emphysema (1), pleural effusion (1), pneumothorax (1) and acute diverticulitis (1). None of the above-mentioned complications were life-threatening

### Comparison of neurological outcomes between thymectomy and conservative LOMG groups

**Fig. 1:**
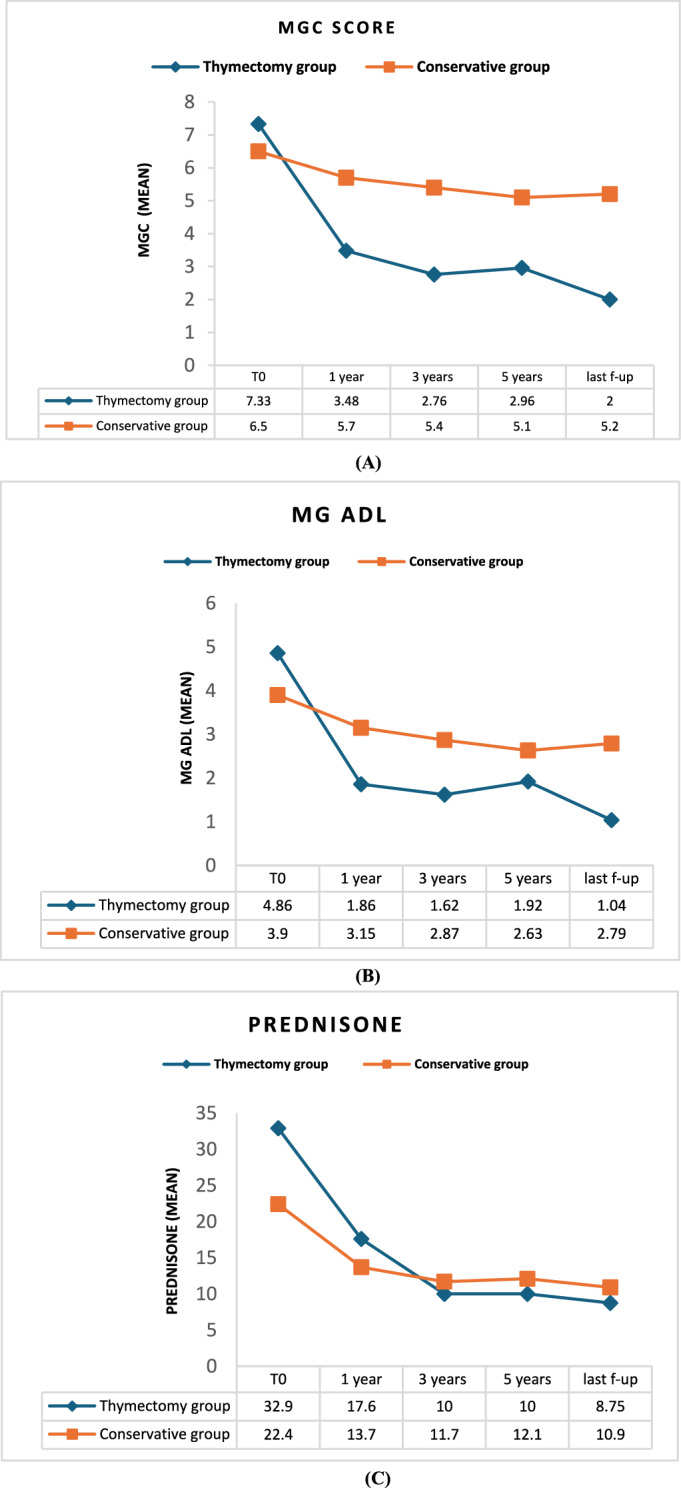
Prednisone dosage, MGC score and MG ADL decline during the follow-up. Percentage changes in MGC (A), MG ADL scales (B) and prednisone dosage (C) were more prominent in the thymectomy group (*p* < 0.001 – Mann–Whitney Test). *MG ADL* Myasthenia Gravis Activities of Daily Living, *MGC score* Myasthenia Gravis Composite score, *SD* Standard deviation, *IQR* Interquartile range, *CSR* Complete stable remission, *PR* Pharmacological remission, *ns* Not significant

Prednisone dosage, MGC, and MG ADL scores decreased during follow-up in both groups but to a greater extent in the thymectomy group (*p* < 0.001, Fig. 1 and Table 3**)**. Moreover, at the last follow-up, the proportion of patients on immunosuppressive treatment remained higher in the conservative group (*p* = 0.001). No differences were observed in the requirement for rescue therapy (IVIg/PLEX) between the two groups during the follow-up.

**Table 3:** Comparison of neurological outcomes at last follow-up

	Thymectomy group *n* = 86	Conservative group *n* = 40	*p* value*
Δ prednisone dosage median (IQR)	− 17.5 (21.25) mg/die	− 3.75 (20.3) mg/die	**0.001**
Δ MG ADL median (IQR)	− 4 (3)	− 0 (2)	**< 0.001**
Δ MGC score median (IQR)	− 4 (6)	− 0.5 (6)	**< 0.001**
Immunosuppressants n (%)	11 (12.6%)	15 (37.5%)	**0.001**
IVIg/PLEX n (%)	7 (8%)	6 (15%)	ns
MGFA classification at last follow-up n (%)			
0	43 (49.4%)	7 (17.5%)	**< 0.001**
1	9 (10.3%)	5 (12.5%)	ns
2A	20 (23%)	7 (17.5%)	ns
2B	5 (5.7%)	8 (20%)	**0.014**
3A	2 (2.3%)	8 (20%)	**< 0.001**
3B	8 (9.2%)	3 (7.5%)	ns
4B	0	2 (5%)	**0.036**

Δ Delta (i.e., change from baseline to last follow-up), *MG ADL* = Myasthenia Gravis Activities of Daily Living; *MGC score* = Myasthenia Gravis Composite score; *SD* Standard deviation, *IQR* Interquartile range, *ns* Not significant, *MGFA-PIS* Myasthenia Gravis Foundation of America post-intervention status, *CSR* Complete stable remission, *PR* Pharmacological remission, *MM* Minimal manifestation, *U* Unchanged, *W* Worse, *f-up* Follow-up, *MGFA* Myasthenia Gravis Foundation of America (MGFA = 0 was assigned to asymptomatic patients)

^*^Chi-square test for categorical variables, Mann–Whitney test for continuous variables

The greatest decrease in prednisone dosage and the most pronounced clinical improvement (as indicated by the decline in MG-ADL and MGC scales) were observed in the thymectomy group in the first year after surgery. Since then, patients continued to experience further albeit slower clinical improvement.

The median duration of the follow-up was 36 (54) months [median (IQR)] with 49 patients out of 127 with a follow-up of 60 months (5 years) or longer.

At the last follow-up, when evaluating disease severity according to MGFA classification, the thymectomy group exhibited a higher proportion of patients with mild disease or asymptomatic status compared to the conservative treatment group compared to the conservative group (Table 3).

**Fig. 2:**
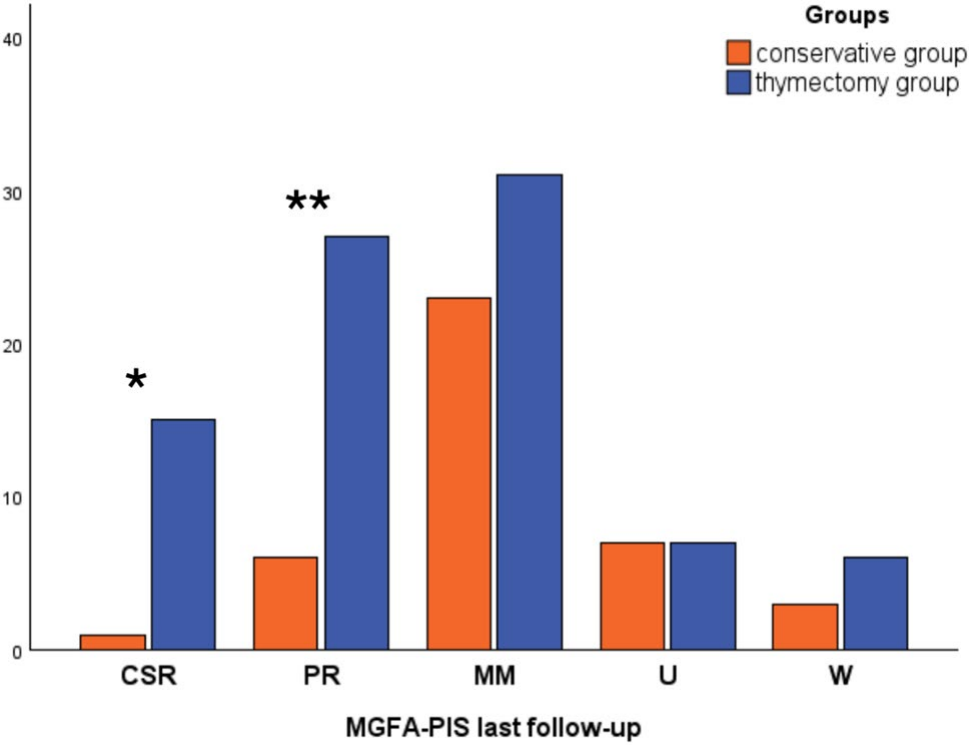
MGFA-PIS at last follow-up. Chi-square test for categorical variables. *p = 0.014 **p = 0.002. *MGFA-PIS* Myasthenia gravis foundation of America post-intervention status, *CSR* Complete stable remission, *PR* Pharmacological remission, *MM* Minimal manifestation, *U* Unchanged, *W* Worse

According to the MGFA-PIS at the last follow-up, 102 (81.6%) patients of the entire cohort had a positive outcome, including minimal manifestation status (CSR + PR + MM), with 16 patients (12.8%) achieving CSR and 32 (25.2%) patients achieving PR. The proportion of patients achieving disease remission (both CSR and PR) was higher in the thymectomy group (data shown in Fig. 2).

**Fig. 3:**
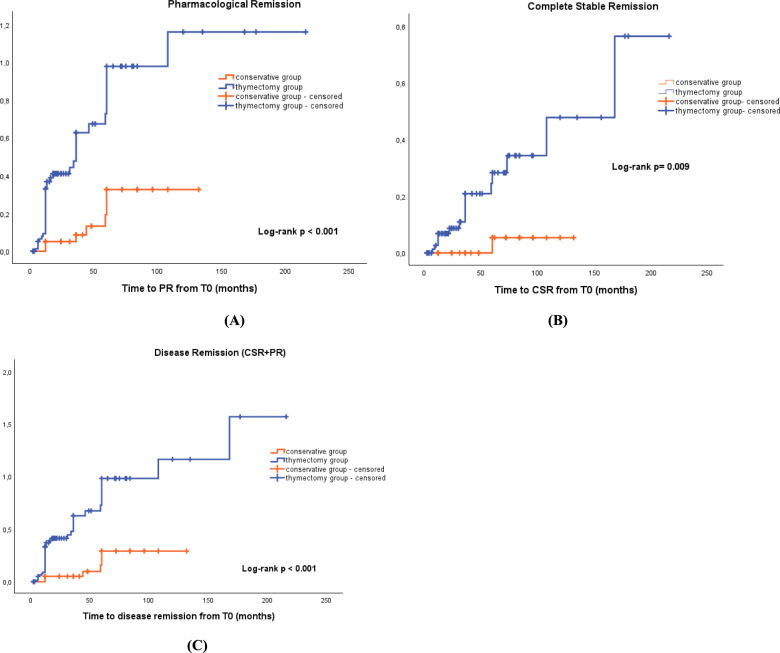
Kaplan–Meier curves for cumulative incidence of PR, CSR and disease remission. Kaplan–Meier curves of cumulative incidence of PR (**A**), CSR (**B**) and disease remission (**C**) in the LOMG patients who underwent thymectomy (thymectomy group) and those who did not (conservative group). *CSR* Complete stable remission, *PR* Pharmacological remission, *LOMG* Late-onset myasthenia gravis

Kaplan–Meier analysis was performed to examine the cumulative incidence curves of PR, CSR and disease remission (PR + CSR) between patients who underwent thymectomy and those who received only medical treatment. Kaplan–Meier plots between the two groups were compared using the log-rank test (Fig. 3). The cumulative incidence of PR and CSR was higher among the thymectomy group than among the conservative group (*p* < 0.001 and *p* = 0.009, respectively). The thymectomy group had a 9.17-fold [hazard ratio (HR) = 9.17, 95% confidence interval (CI) 1.21–69.49] higher chance of achieving CSR and a 3.86-fold (HR = 3.86, 95% CI 1.72–8.66) higher chance of achieving PR compared to the conservative group. Considering PR and CSR together as Disease Remission, the thymectomy group had a 4.48-fold [hazard ratio (HR) = 4.48, 95% confidence interval (CI) 1.89–10.60] higher chance of achieving clinical remission than the group of patients treated with medical therapy alone.

The independent effect of thymectomy on attaining Disease Remission was further evaluated using the Cox proportional hazards regression model to adjust for the confounding effects of other variables (Table 4). After adjustment for age, sex, immunotherapy, and disease severity, the thymectomy group still had a 3.25-fold (HR = 3.25, 95% CI 1.308–8.075) increased probability of achieving disease remission than the conservative group.

**Table 4:** Cox regression model

					95,0% CI for Exp (B)	
	B	SE	Sign	Exp(B)	Inf	Upp
Sex	0.068	0.354	0.849	1.070	0.535	2.141
Age at onset	0.025	0.021	0.229	1.025	0.985	1.067
MGFA at onset	− 0.416	0.128	**0.001**	0.660	0.514	0.848
MGC at baseline	0.029	0.049	0.560	1.029	0.9235	1.133
MG ADL at baseline	− 0.044	0.086	0.606	0.957	0.808	1.133
Immunosuppressants at baseline	− 0.517	0.492	0.293	0.596	0.227	1.564
Prednisone dosage at baseline	0.008	0.010	0.399	1.008	0.989	1.028
Thymectomy	1.179	0.464	**0.011**	3.250	1.308	8.075

The Cox proportional hazards regression model was used to estimate the effect of thymectomy on achieving disease remission after adjustment for age, sex, and disease severity at onset, disease severity at baseline and prednisone and immunosuppressants use at baseline

*MGFA* Myasthenia gravis foundation of America, *MG ADL* Myasthenia gravis activities of daily living, *MGC score* Myasthenia gravis composite score

Sex, age at onset, and disease severity at baseline were not correlated with achieving disease remission, nor was the use of prednisone or immunosuppressants at baseline.

However, MGFA classification at onset was found to be a negative predictive factor for achieving disease remission. Indeed, having a higher MGFA classification (i.e., greater disease severity) at onset was associated with poorer outcomes.

### Focus on thymic histology and surgical approach

We also analyzed the clinical and demographic features at baseline of the 87 patients who underwent thymectomy depending on histopathologic results finding no relevant differences, except for a higher proportion of males in the thymic atrophy group (*p* = 0.044; data not shown). During follow-up, the MG ADL and MGC scales showed a greater decrease in patients with thymic hyperplasia (p 0.022 and 0.042, respectively), whereas no differences were found in the median decrease in prednisone dosage between patients with thymic hyperplasia and those with involuted thymus (Supplementary Table 1). Nevertheless, when comparing neurological outcomes based on thymic pathology, we did not appreciate any difference in the rates of disease re mission (both PR and CSR) after thymectomy (log-rank test *p* = 0.433, see Supplementary Fig. 1).

In our cohort, we observed no differences in the occurrence of post-operative complications based on the surgical approach. Most importantly, the surgical approach (robotic surgery vs sternotomy) did not influence neurological outcomes either (data not shown). We did not observe a correlation between preoperative disease duration and disease remission after thymectomy.

### Response to thymectomy in very late-onset myasthenia gravis (VLOMG) subgroup

In our cohort of LOMG patients, we also identified 26 patients with VLOMG (i.e., age at onset > 65 years). Twelve VLOMG patients underwent thymectomy, while 14 were treated with medical therapy alone. The two groups differed at baseline in terms of age at onset, which was higher in the conservative group, while ocular symptoms at onset and median prednisone dosage at baseline were greater in the thymectomy group (see Supplementary Table 2). We also investigated neurological outcomes of thymectomy in this subgroup and observed that even in VLOMG thymectomy was effective in achieving disease remission (see Supplementary Fig. 2 and Supplementary Table 3) compared to medical therapy alone. The Cox proportional hazards regression model again confirmed the effect of thymectomy on achieving disease remission after adjusting for age, sex, and disease severity at onset, disease severity at baseline, and baseline use of prednisone and immunosuppressants. VLOMG patients who underwent thymectomy had an 8.85-fold (HR = 8.85, 95% CI 1.05–74.3) increased probability of achieving disease remission compared to the conservative group.

### EOMG versus LOMG patients

At baseline, the two groups showed no differences in MG-ADL, MGC scale scores, or prednisone dosage. When comparing neurological outcomes between EOMG and LOMG patients after thymectomy, no differences were observed in MG-ADL and MGC scores at the last follow-up, nor in the median delta change of scale scores or prednisone daily dosage between baseline and the last follow-up. However, the median prednisone dosage at the last follow-up in EOMG patients was lower [5 (15) mg/die; *p* = 0.025] than the steroid dosage in LOMG patients, which was 7.5 (22.5) mg/die [median (IQR)]. No differences were observed in the use of immunosuppressants at last follow-up.

Regarding MGFA-PIS at last follow-up, EOMG patients achieved CSR more frequently (*p* = 0.040), while LOMG patients achieved more frequently PR after thymectomy (*p* = 0.018—see Supplementary Table 4).

When comparing the cumulative incidence of disease remission (PR + CSR) between EOMG and LOMG patients, no differences were observed (see Supplementary Fig. 3). Notably, LOMG patients reached PR earlier than EOMG patients (*p* = 0.003), with a median time to PR of 21 (38) months versus 36 (48) months [median (IQR)]. Similarly, LOMG patients achieved disease remission (PR + CSR) faster than EOMG patients (*p* < 0.001).

## Discussion and conclusions

So far, only three retrospective studies have directly compared the effectiveness of thymectomy versus conservative treatment for non-thymomatous LOMG [12–14]. Romi et al. found no difference in rates of disease remission between thymectomized (*n* = 22) and non-thymectomized (*n* = 21) LOMG patients, while observing a correlation between seropositivity for anti-titin antibodies and poorer outcomes [12]. In 2007, Kawaguchi et al. investigated the effectiveness of thymectomy in a cohort of 34 LOMG patients, suggesting a potential benefit of the procedure versus standard conservative treatment [13]. However, the authors assessed neurological outcomes of thymectomy using the MGFA classification, a fact that hinders a proper comparison of their findings with other studies, including the present study. Finally, Kim et al. directly compared the effects of thymectomy versus conservative treatment in a group of 139 LOMG patients (34 thymectomized and 105 non-thymectomized, respectively) finding that the cumulative incidence of both PR and CSR was higher in the thymectomy group than in the medical treatment group. After adjustment for age, sex, and disease severity, the thymectomy group still had a 2.22-fold (HR = 2.22, 95% CI 1.01–4.80) increased chance of achieving PR than the conservative group (although no differences remained in achieving CSR) [14].

Our population of 87 LOMG patients who underwent thymectomy is the largest analyzed to date for clinical outcomes and compared to a control group treated with medical therapy alone. The present study demonstrated the beneficial effect of thymectomy in LOMG patients compared to medical treatment alone. Indeed, the thymectomy group of our LOMG cohort had a 3.25-fold (HR = 3.25, 95% CI 1.308–8.075) increased probability of achieving disease remission (CSR + PR) than the conservative group, after adjustment for age, sex, immunotherapy, and disease severity. Furthermore, the thymectomy group experienced a greater clinical improvement (as evidenced by MG-ADL and MGC scores decline) and a greater steroid-sparing effect after thymectomy compared to the conservative group, without ongoing immunosuppressive treatment. In this regard, the proportion of patients receiving immunosuppressants was higher in the conservative group both at baseline and at the last follow-up.

The procedure was generally safe, with few post-operative complications, regardless of the surgical approach, and no serious or life-threatening adverse events were recorded. Moreover, the surgical approach (robotic surgery vs sternotomy) did not influence neurological prognosis after the procedure.

Whether thymic histology correlates with response to thymectomy in MG is still unclear. One of the main reasons why thymectomy has been considered less effective in LOMG is the higher prevalence of thymic atrophy in this population. This results in the generation of self-reactive T cells and the production of self-perpetuating pathogenic AChR antibodies outside the thymus, likely explaining the relative lower effectiveness of thymectomy in LOMG [9].

Studies on predictors of outcome and remission after thymectomy have shown that histology can also influence the outcome, with thymic hyperplasia appearing to be associated with a higher likelihood of disease remission after the intervention [29–32]. Uzawa et al. retrospectively reviewed 2 year post-thymectomy prognoses in 39 consecutive LOMG patients. In their cohort, LOMG patients with thymic hyperplasia showed a higher rate of remission and received lower prednisolone doses compared to patients with involuted thymuses [33]. The authors concluded that in generalized LOMG patients presenting with thymic hyperplasia on chest CT, thymectomy could be considered as a principal treatment for MG. However, Chen et al. observed that patients with atrophic thymus had a similar post-operative prognosis to those with hyperplastic thymus, suggesting that surgical therapy should also be considered for the former subset. They proposed that the atrophic thymus may contribute to the progression of MG probably due to the presence of activated B cells even in the thymic tissue undergoing involution [34]. Nakahara et al. also compared clinical features and post-operative prognoses among three different thymic histologies (thymic hyperplasia, thymoma, and thymic atrophy) and found that the clinical course of MG patients with atrophic thymus after thymectomy was better than that of those with hyperplasia or thymoma [35]. In the current study, histological examination revealed thymic atrophy in 51.6% of patients, while thymic hyperplasia was found in 31.5% of patients. During follow-up, the MG-ADL and MGC scales showed a greater decrease in patients with thymic hyperplasia, while no differences were found in the percentage decrease in prednisone dosage between patients with thymic hyperplasia and those with involuted thymus. Nevertheless, when comparing neurological outcomes based on thymic pathology, we did not appreciate any difference in the rates of disease remission after thymectomy.

In our cohort, when evaluating possible predictive factors for disease remission, sex, age at onset, disease severity at baseline, and immunomodulatory therapies used were not correlated with achieving disease remission, while the MGFA classification at onset was found to be a predictive factor for poorer outcomes. This finding is in line with previous reports showing better outcomes after thymectomy in patients with mild generalized disease pre-surgery according to MGFA classification [36, 37]. Among the thymectomy group, we did not observe a relationship between the timing of the intervention and neurological outcome. This finding is in contrast with what was reported from the latest meta-analysis on the topic [16] showing a higher response rate in patients with a preoperative disease duration of less than three years, but it could be explained by the fact that most of patients in the present study underwent thymectomy within 24 months from disease onset.

Although there is no set age limit for thymectomy, most experts do not recommend surgery for most patients > 60 years of age, based on a concern that the risks of thymectomy outweigh the potential benefits at this age, unless a thymoma is present. With that respect, we analyzed neurological outcomes of thymectomy compared with conservative treatment even in the VLOMG subgroup. Our data seem to suggest that thymectomy is effective in achieving disease remission, even in this subgroup, conferring a8.85-fold (HR = 8.85, 95% CI 1.05–74.3) increased likelihood of achieving remission compared to medical therapy alone. This result should still be interpreted with caution given the small sample size of the VLOMG subgroup. However, this observation aligns with the findings of Lin et al. in their monocentric retrospective study on robotic thymectomy in MG patients older than 60 years at onset. Although lacking a control group of elderly patients who did not undergo thymectomy, they found robotic thymectomy to be safe and effective [31].

Regarding the comparison of long-term effectiveness of thymectomy between EOMG and LOMG patients, data available from the nine studies conducted to date provided controversial results, with some reports favoring EOMG over LOMG patients [30, 38–41] and others finding no differences between the two groups [31, 42–44].

In line with previous studies, we also compared post-thymectomy outcomes of our LOMG cohort with a group of EOMG patients, the only subgroup of MG patients in whom thymectomy has been proven effective in a randomized trial [10, 11]. We did not observe any differences in MG-ADL and MGC scores at the last follow-up, nor in the median change of scale scores or prednisone daily dosage following thymectomy between EOMG and LOMG patients. However, the median prednisone dosage at the last follow-up in EOMG patients was lower than that of LOMG patients, a finding likely attributable to the higher rates of CSR observed in EOMG patients.

Regarding MGFA-PIS at last follow-up, a higher proportion of EOMG patients achieved CSR, while LOMG patients achieved PR more frequently after thymectomy. However, when comparing the cumulative incidence of disease remission (PR + CSR) between EOMG and LOMG patients, no differences were observed. Notably, LOMG patients reached PR earlier than EOMG patients (*p* = 0.003), with a median time to PR of 21 (38) months versus 36 (48) months [median (IQR)]. Similarly, LOMG patients achieved disease remission (PR + CSR) faster than EOMG patients (*p* < 0.001). This aspect is particularly relevant in this population, considering the need for special attention to early treatment choices in LOMG patients due to the higher incidence of life-threatening events early in the course of the disease compared to EOMG [5].

The present study has several limitations. The retrospective design makes it sensitive to several biases, selection ones above all, and this may limit the generalizability of the results.

The principal source of selection bias lies in the clinical indication for either surgical intervention or conservative management. Within our cohort of LOMG, the predominant factors guiding the recommendation for thymectomy included inadequate disease control, the therapeutic objective of minimizing long-term exposure to steroid and immunosuppressive agents, and, in certain cases, the radiological detection of indeterminate thymic nodules on chest computed tomography (CT) imaging. Conversely, in the conservatively managed group, the decision to forego surgical intervention was primarily based on a comprehensive clinical assessment weighing comorbid conditions, peri-operative anesthetic risk, and overall disease burden. In a minority of cases, patient refusal also contributed to the choice of conservative treatment.

Our study is a tertiary clinic-based and might not fully reflect the local population due to a referral bias, as very elderly patients, for instance, may not be referred to tertiary hospitals for further treatment in a specialized unit. Moreover, the thymectomy and conservative groups were not homogeneous in terms of age at onset. Similar to Kim et al. [14] and Kawaguchi et al. [13], age at onset was younger in the thymectomy group than in the medical treatment group. This observation is likely attributable to the reluctance of neurologists to propose thymectomy to elderly patients, considering their higher surgical risk. However, in our analysis age at onset was not found to be a confounding factor for achieving remission or for the other long-term clinical and therapeutic outcomes. More critically, the two groups were also not comparable in terms of MGFA classification at onset, which was more severe in the conservative group and appeared to predict poorer outcomes. Nonetheless, regression analysis indicated that this aspect did not seem to attenuate or negate the observed benefit of thymectomy.

Finally, the presence of a placebo effect in the thymectomy group cannot be entirely excluded; however, the extended post-operative follow-up period strengthens the inference that, if present, such an effect is likely to be minimal in clinical relevance. One of the main strengths of the study is certainly the sample size, which is considerably larger than that in former published works, as well as the long follow-up period after intervention and the combined use of MG-ADL, MGC scores, MGFA-PIS and daily steroid dosage to evaluate neurological outcomes.

Despite the limitations previously highlighted, our findings suggest that thymectomy may be a feasible and potentially beneficial therapeutic option in selected patients within this MG subgroup. In our cohort, thymectomy appeared to be associated with a higher probability of achieving both complete clinical and pharmacological remission without ongoing immunosuppressive therapy in LOMG patients, compared to medical therapy alone. These observations suggest that age alone might not necessarily preclude surgical consideration. Nonetheless, given the increased burden of comorbidities and potential surgical risks associated with aging, thymectomy should be evaluated cautiously, guided by individual patient profiles and multidisciplinary assessment. Further prospective, randomized controlled studies are needed to more conclusively inform treatment strategies and address the therapeutic needs of this growing MG population.

## Supplementary Information

Below is the link to the electronic supplementary material.

Supplementary file1 (PDF 44 KB)

Supplementary file2 (PDF 26 KB)

Supplementary file3 (PDF 159 KB)

Supplementary file4 (DOCX 28 KB)

## Data Availability

Data can be made available on reasonable request. Requests should be directed to the corresponding author.
