# Supplementary material for: Long-term effectiveness of thymectomy in late-onset myasthenia gravis
Source: J Neurol. 2025 Oct 21;272(11):714. doi: 10.1007/s00415-025-13424-2 (PMC12540559; doi:10.1007/s00415-025-13424-2)
Supplement: Supplementary file 1 — Supplementary file1 (PDF 44 KB) [file 415_2025_13424_MOESM1_ESM.pdf]

Supplementary Figure 1 - Cumulative incidence of disease remission after thymectomy depending on thymus histology

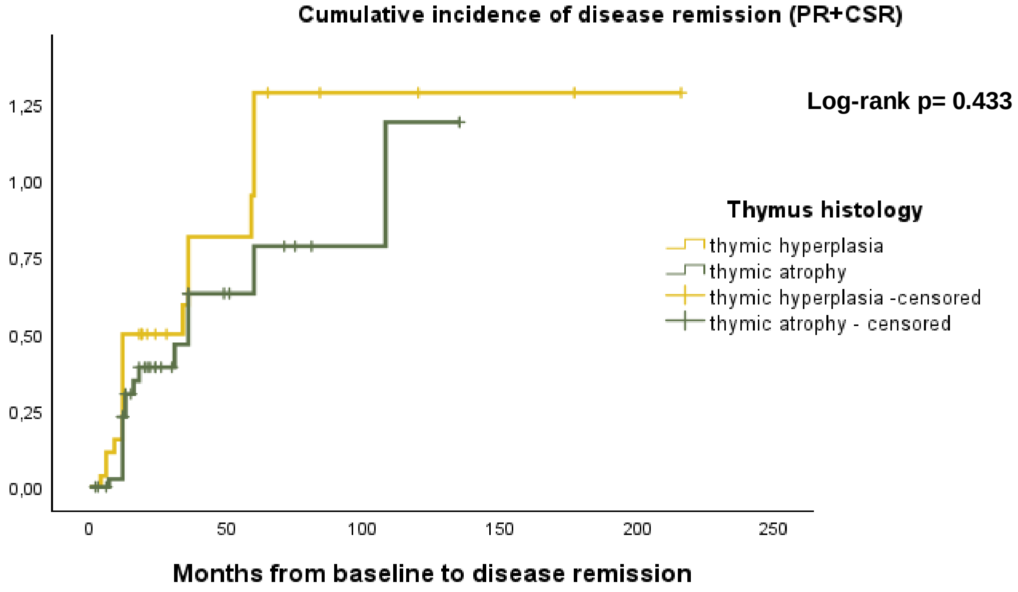

Kaplan-Meier curves of patients reaching clinical remission with thymic hyperplasia or thymic atrophy on histological examination of thymic tissue.
List of abbreviations: CSR= complete stable remission; PR= pharmacological remission.
