# Supplementary material for: Long-term effectiveness of thymectomy in late-onset myasthenia gravis
Source: J Neurol. 2025 Oct 21;272(11):714. doi: 10.1007/s00415-025-13424-2 (PMC12540559; doi:10.1007/s00415-025-13424-2)
Supplement: Supplementary file 2 — Supplementary file2 (PDF 26 KB) [file 415_2025_13424_MOESM2_ESM.pdf]

Supplementary Figure 2. Kaplan-Meier curves for cumulative incidence of disease remission in VLOMG

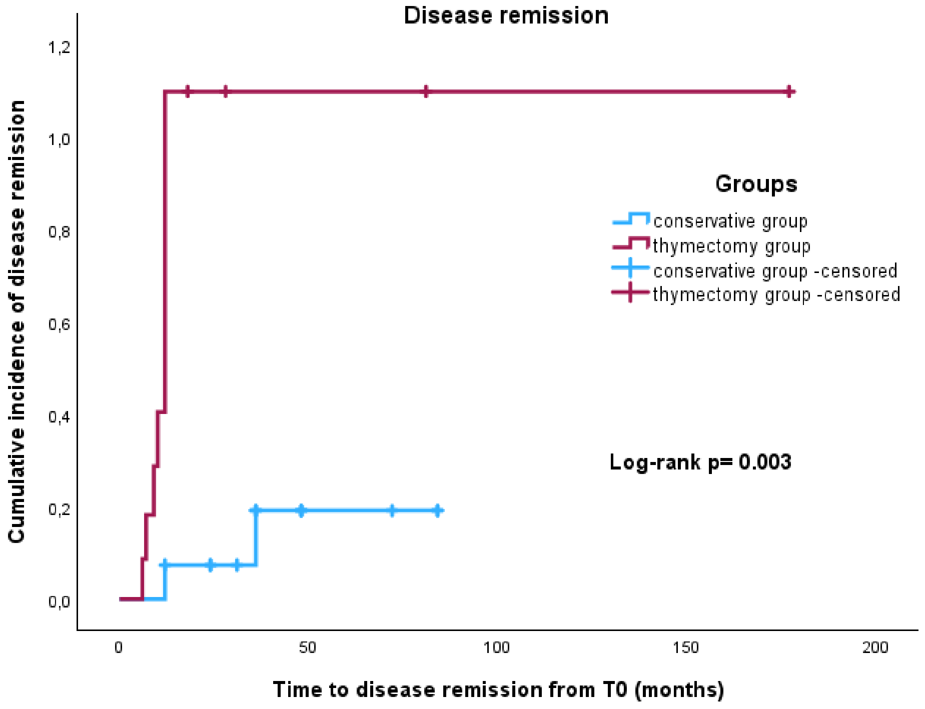

Kaplan-Meier curves of cumulative incidence of disease remission (PR+CSR) in the VLOMG patients who underwent thymectomy (thymectomy group) and those who did not (conservative group).
List of abbreviations: CSR= complete stable remission; PR= pharmacological remission; VLOMG= very-late-onset Myasthenia Gravis.
