# Supplementary material for: Long-term effectiveness of thymectomy in late-onset myasthenia gravis
Source: J Neurol. 2025 Oct 21;272(11):714. doi: 10.1007/s00415-025-13424-2 (PMC12540559; doi:10.1007/s00415-025-13424-2)
Supplement: Supplementary file 3 — Supplementary file3 (PDF 159 KB) [file 415_2025_13424_MOESM3_ESM.pdf]

Supplementary Figure 3. Kaplan-Meier curves for cumulative incidence of PR, CSR and disease remission

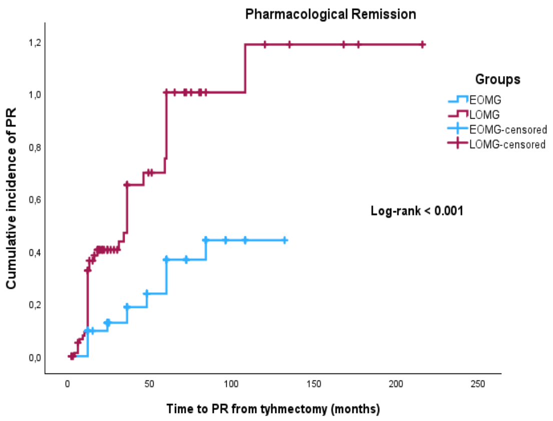

(A)

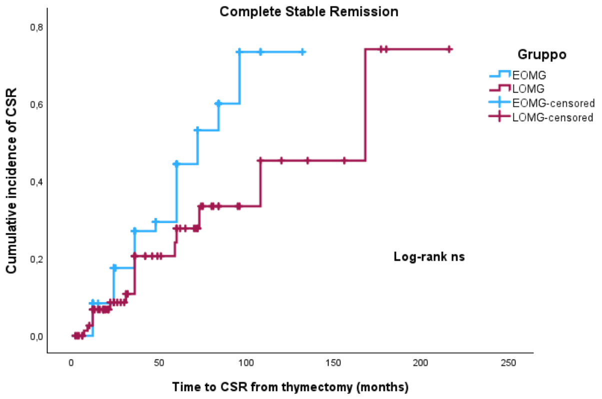

(B)

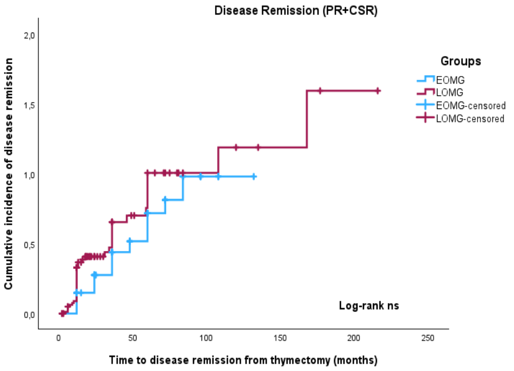

(C)

Kaplan-Meier curves of cumulative incidence of PR (A), CSR (B) and disease remission (C) in the LOMG and EOMG patients who underwent thymectomy.

List of abbreviations: CSR= complete stable remission; PR= pharmacological remission; LOMG= late-onset Myasthenia Gravis; EOMG = early-onset Myasthenia Gravis
