# Supplementary material for: Long-term effectiveness of thymectomy in late-onset myasthenia gravis
Source: J Neurol. 2025 Oct 21;272(11):714. doi: 10.1007/s00415-025-13424-2 (PMC12540559; doi:10.1007/s00415-025-13424-2)
Supplement: Supplementary file 4 — Supplementary file4 (DOCX 28 KB) [file 415_2025_13424_MOESM4_ESM.docx]

**Supplementary Material**

**Supplementary Table 1 -Comparison of neurological outcomes of thymectomy depending on thymic pathology**

|  | Thymic hyperplasia  n=31 | | | | Thymic atrophy n=45 | p value* | |
| --- | --- | --- | --- | --- | --- | --- | --- |
| Δ prednisone dosage median (IQR) | | - 22.5 (20) mg/die | | | - 17.5 (21.25) mg/die | ns | |
| Δ MG ADL median (IQR) | | - 4 (3) | | | - 3 (3) | **0.022** | |
| Δ MGC score median (IQR) | | - 7 (5) | | | - 4 (5) | **0.042** | |
| Disease Remission n (%)  CSR  PR | | | 8 (25.8%)  17 (54.8%) | 6 (13.3%)  17 (37.8%) | | | ns  ns |

* Chi-square test for categorical variables, Mann–Whitney test for continuous variables

List of abbreviations: Δ = delta (i.e., change from baseline to last follow-up); MG ADL= Myasthenia Gravis Activities of Daily Living; MGC score= Myasthenia Gravis Composite score; SD=standard deviation; IQR= interquartile range; ns= not significant.

**Supplementary Table 2. Comparison of baseline characteristics of thymectomy and conservative VLOMG cohorts**

|  | Thymectomy group n=12 | Conservative group n=14 | p value* |
| --- | --- | --- | --- |
| Sex n (%)  Female  Male | 6 (50%)  6 (50%) | 4 (28.6%)  10 (71.4%) | ns |
| Age at onset median (IQR) | 70.5 (7) years | 77.5 (8) years | **<0.001** |
| MGFA classification at onset n (%)  1  2A  2B  3A  3B  4B  5 | 3 (25%)  0  5 (41.7%)  0  4 (33.3%)  0  0 | 0  0  8 (57.1%)  0  4 (28.6%)  1 (7.1%)  1 (7.1%) | **0.047**  ns  ns  ns  ns  ns  ns |
| MG treatment at baseline n (%)  Prednisone  Immunosuppressants | 12 (100%)  0 | 13 (92.8%)  3 (21.4%) | ns  ns |
| Dose of prednisone at baseline median (IQR) | 25 (12.5) mg/die | 13.75 (19.4) mg/die | **0.020** |
| Immunosuppressants at baseline n (%) | 0 | 3 (21.4%) | ns |
| IVIG/PLEX at baseline n (%) | 0 | 4 (28.6%) | **0.044** |
| MG ADL at baseline median (IQR) | 4.5 (3) | 2.5 (5) | ns |
| MGC score at baseline median (IQR) | 6 (7) | 3.5 (8) | ns |
| Comorbidities n (%)  DM  CAD  Cerebrovascular disease  AF  Hypertension  COPD  Osteoporosis  Extra-thymic malignancy  Autoimmune disease | 4 (33.3%)  0  1 (8.3%)  0  4 (33.3%)  1 (8.3%)  3 (25%)  4 (33.3%)  4 (33.3%) | 2 (14.3%)  4 (28.6%)  4 (28.6%)  1 (7.1%)  10 (71.4%)  2 (14.3%)  4 (28.6%)  4 (28.6%)  9 (64.3%) | ns  **0.044**  ns  ns  **0.052**  ns  ns  ns  ns |

* Chi-square test for categorical variables, Mann–Whitney test for continuous variables

Data are shown as mean ± SD or median (IQR) depending on whether the data showed normal distribution.

List of abbreviations: VLOMG= Very Late-Onset Myasthenia Gravis; MGFA= Myasthenia Gravis Foundation of America; MG ADL= Myasthenia Gravis Activities of Daily Living; MGC score= Myasthenia Gravis Composite score; IVIg= Intravenous immunoglobulin; PLEX= plasma exchange; SD=standard deviation; IQR= interquartile range; DM= diabetes mellitus; CAD= coronary artery disease; AF= atrial fibrillation; COPD= Chronic obstructive pulmonary disease.

**Supplementary Table 3 -Comparison of neurological outcomes in VLOMG**

|  | Thymectomy group n=12 | | Conservative group n=14 | p value* | |
| --- | --- | --- | --- | --- | --- |
| Δ prednisone dosage median (IQR) | - 20 (12.5) mg/die | - 17.5 (25) mg/die | | | ns |
| Δ MG ADL median (IQR) | - 3 (4) | - 0 (3) | | | **0.009** |
| Δ MGC score median (IQR) | - 6 (5) | - 0 (9) | | | **0.004** |
| Disease Remission** n (%) | 8 (66.7%) | 2 (14.2%) | | | **0.006** |

* Chi-square test for categorical variables, Mann–Whitney test for continuous variables

** Disease Remission = CSR+PR.

List of abbreviations: Δ = delta (i.e., change from baseline to last follow-up); MG ADL= Myasthenia Gravis Activities of Daily Living; MGC score= Myasthenia Gravis Composite score; CSR= complete stable remission; PR= pharmacological remission; SD=standard deviation; IQR= interquartile range; ns= not significant

**Supplementary Table 4 -Comparison of neurological outcomes at last follow-up**

|  | EOMG group  n=87 | LOMG group  n=86 | | p value* |
| --- | --- | --- | --- | --- |
| MGFA-PIS at last f-up, n (%)  CSR  PR  MM  U  W | 28 (32.2%)  14 (16.1%)  31 (35.6 %)  6 (6.9%)  8 (9.2%) | | 14 (17.5%)  25 (31.3%)  23 (37.5%)  6 (7.5%)  5 (6.3%) | **0.040**  **0.018**  **ns**  **ns**  **ns** |
| Δ prednisone dosage median (IQR) | - 22.5 (25) mg/die | | - 17.5 (20.63) mg/die | **ns** |
| Δ MG ADL median (IQR) | -4 (3) | | - 4 (3) | **ns** |
| Δ MGC score median (IQR) | -6 (5) | | -4 (6) | **ns** |
| Immunosuppressants n (%) | 12 (13.8%) | | 11 (12.6%) | **ns** |
| Prednisone dosage median (IQR) | 5 (15) mg/die | | 7.5 (22.5) mg/die | **0.025** |
| Time to PR median (IQR) | 36 (48) | | 20 (38) | **0.003** |
| Time to disease remission median (IQR) | 36 (48) | | 20.5 (38) | **<0.001** |

* Chi-square test for categorical variables, Mann–Whitney test for continuous variables

List of abbreviations: Δ = delta (i.e., change from baseline to last follow-up); MG ADL= Myasthenia Gravis Activities of Daily Living; MGC score= Myasthenia Gravis Composite score; SD=standard deviation; IQR= interquartile range; ns= not significant; MGFA-PIS= Myasthenia Gravis Foundation of America Post-Intervention Status; CSR=Complete Stable Remission; PR=Pharmacological Remission; MM= Minimal Manifestation; U=unchanged; W=worse; f-up= follow-up.
